# New profit models for property management in the post-pandemic era: A study on consumer attitudes towards community value-added services

**DOI:** 10.1371/journal.pone.0314328

**Published:** 2024-12-02

**Authors:** Meng Lin, Weidong Li, Yukun Cao, Yujuan Gao

**Affiliations:** 1 Heilongjiang University, Harbin, China; 2 Capital University of Economics and Business, Beijing, China; 3 Northeast Forestry University, Harbin, China; University of Georgia, UNITED STATES OF AMERICA

## Abstract

The COVID-19 pandemic, which emerged in 2020, has had a tremendous impact on various industries and the daily lives of the general populace, thereby contributing to increasing uncertainty in business competition. Property management enterprises, despite witnessing a mostly stagnant expansion and enhancement of their existing operations, have, owing to the unique nature of their services, continued to experience a relatively positive competitive environment. Notably, the recent emphasis in property management companies has been on the development of value-added services at the community level, which represents a critical avenue for securing a competitive edge by addressing consumer demands. This study, grounded in the ABC theory and the theory of planned behavior, seeks to analyze consumer attitudes towards the provision of value-added services by property management firms, including facets such as community healthcare services, community lifestyle amenities, community educational offerings, and personalized services. Moreover, the study delves into the mediating role played by satisfaction with fundamental property management services. The empirical findings emphasize the significant enhancement in consumer receptivity to property management firms’ value-added services, particularly in the domains of community healthcare, lifestyle amenities, education, and personalized services. In addition, it highlights the indirect effect of property owners’ contentment with essential property management services on their perspectives concerning the delivery of value-added services by property management firms. The implications of these research conclusions bear significant relevance for organizations engaged in offering such services.

## Introduction

Traditionally, property management refers to the activities of property owners and property service enterprises in accordance with the contract, in which the property service enterprise repairs, maintains, and manages the house and supporting facilities, and maintains the environmental hygiene and order within the management area [1–3]. Property management enterprises (property companies) are service-oriented enterprises established in accordance with relevant laws. Property management companies accept the commission of property owners to implement specialized management of properties within a specific area and receive corresponding compensation. In the backdrop of the COVID-19 pandemic, a significant transformation has swept through various housing sectors, reshaping production, operations, and everyday life. Due to extensive responsibilities in epidemic prevention and control, the property management company’s core business has stalled. Nevertheless, the idiosyncratic nature of property-related services has endowed the property management sector with a relatively favorable competitive landscape. Amid the prevailing conditions marked by pandemic-induced uncertainties, the service requisites of property owners have diversified substantially, with higher requirements for service quality. Traditional foundational services have lost their capacity to cater to the evolving demands of property owners, thereby accentuating the necessity of identifying novel avenues for economic growth in property management firms. Fundamental property management services, characterized by slender profit margins and high degrees of uniformity, have hitherto relied primarily on the expansion of managed properties to fuel growth, often falling short in achieving economies of scale. In contrast, the burgeoning domain of community value-added services has yielded lucrative profit margins and differentiation, thereby facilitating rapid profit growth. These services are additional value services provided by property enterprises beyond basic services, such as community asset management, home life services, and commercial service operations [4, 5]. Additionally, the adoption rates of community value-added services continue to surge, with communities themselves witnessing significant population influx. Hence, as the scope of managed properties expands for property service enterprises, the consumer base for community value-added services correspondingly burgeons, leading to economies of scale and a sustained uptick in operational revenue for these firms.

Numerous strides have been made in community value-added services research. Existing studies mostly focus on the effect of value-added services on profit margins increase, resident satisfaction enhancement, as well as the specific projects and forms of value-added services implementation by most property management companies. Chen posited that, driven by rising costs and increased operational expenditures, numerous property management enterprises are now venturing into the domain of community value-added services to elevate service quality and optimize profits [6]. In response to the pandemic’s influence, which necessitated measures such as confined community management and home quarantine, the implementation of community value-added services has emerged as a means to enrich the quality of life for property owners while aligning with industry progress. Since the emergence of the COVID-19 pandemic at the close of 2019, property management’s critical role in epidemic prevention and control has prompted a societal reassessment of the property management sector [7]. This enhanced public standing has created an advantageous backdrop for property service firms to carry out on community value-added services. These services are increasingly contributing to profits and have evolved into a vital facet of property companies’ operations, with diversified activities spotlighting the capability of property service organizations [8, 9]. The share of revenue derived from value-added services is on the rise, marking a sustained growth and establishing itself as a crucial avenue for elevating resident contentment and instilling confidence in the capital market. The specific value-added service offerings have significant effect on property management organizations. If the services rendered fail to effectively meet the requirements of property owners, they will not contribute to satisfaction or generate profits. Huang asserts that property companies can heighten resident satisfaction and amplify profits by offering diverse value-added services such as aesthetically pleasing residences, community intelligence, and innovative business initiatives [10]. Huang and Zeng propose that community value-added service initiatives consist of child transportation to schools, accompanying the elderly to medical appointments, financial advisory, education and training, and job placement [11]. Leveraging internet technology, property firms can offer an array of value-added services to users, thus catering to diverse resident needs while concurrently opening up new revenue streams. Read and Carswell indicate that property employees are now engaged in activities beyond traditional tasks, including budget preparation, financial analysis, market research, contract negotiation, capital project evaluation, and developing corporate social responsibility and environmental sustainability platforms [4]. Chen highlights the significance of personalized service experiences and other deeply customized offerings [12]. He posits that property service enterprises can significantly boost resident contentment and cultivate a positive corporate image by delivering value-added services, thus bolstering economic gains. Value-added services consist of a spectrum of service categories, spanning intermediary services, household upkeep services, maintenance services, and extended service ventures such as personalized service experiences.

This study seeks to probe homeowners’ perspectives concerning property management firms providing community value-added services, with the objective of understanding the level of acceptance accorded to such services. The research serves two principal objectives in the operational domain of property management companies. First and foremost, it strives to offer strategic guidance for property management firms in formulating plans for community value-added services. Invariably, sound business decisions hinge on comprehensive market research. The primary objective of this study offers the foundational underpinnings for decision-making in property management enterprises. The viability of new business ventures is inextricably correlated to the presence of a market, customers, and the acceptance of products and services by these customers. Secondly, subsequent to the attainment of the primary aim, this study seeks to attain an accurate understanding of the specific avenues for property management firms to foster community value-added services in the foreseeable future.

Based on the research of scholars, this study delves into several key facets. Firstly, it explores consumers’ perceptions of property management firms offering supplementary services in the post-COVID-19 landscape. Secondly, it conducts consumer surveys in various business sectors, including healthcare, lifestyle, education, and personalized services, to assess the effect of these offerings on consumer sentiments. Finally, drawing upon empirical analyses, it offers recommendations for the advancement of value-added property services.

## Theoretical foundation

### ABC theory

**Fig 1:**
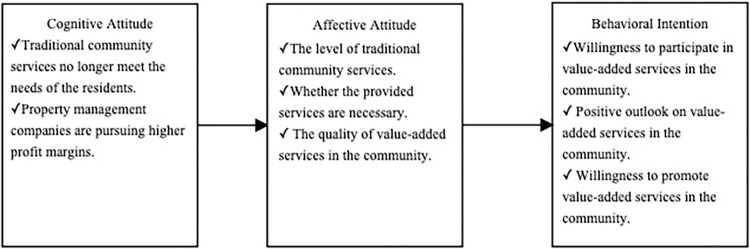
ABC theory model.

The ABC model (Fig 1), proposed by Rosenberg et al., represents a theoretical framework for understanding consumer attitudes [13]. This model posits that consumer attitudes consist of three integral components: cognition, affect, and behavioral intention. Cognition signifies consumers’ knowledge, affect represents their subjective sentiments, and behavioral intention refers to their actions or behavioral tendencies [14]. Cognition serves as the foundational aspect, affect functions as a moderating factor, and behavioral intention refers to the outcome. Prior to making decisions, consumers must possess a certain degree of cognitive understanding. Thereafter, they form subjective evaluations and generate affective responses based on the information and knowledge they have accumulated, leading to specific behavioral intentions [15]. In the aftermath of the pandemic, traditional community services in property management could no longer meet homeowners’ needs, and the profitability of these services dwindled. Therefore, property management companies have been compelled to expand into value-added services to enhance profit margins. Such services can effectively cater to the requirements of homeowners and property management organizations alike. It is necessary for homeowners to acknowledge that the emergence of these value-added services is an inevitable consequence of societal development and transformation. Once homeowners acknowledge the existence of these services, they will develop corresponding affective attitudes. A positive affective attitude towards community value-added services is likely to result in a favorable perception of such offerings and increased participation in services provided by property management companies. Conversely, a negative attitude may lead to opposition against property management companies’ provision of value-added services.

### Theory of planned behavior

**Fig 2:**
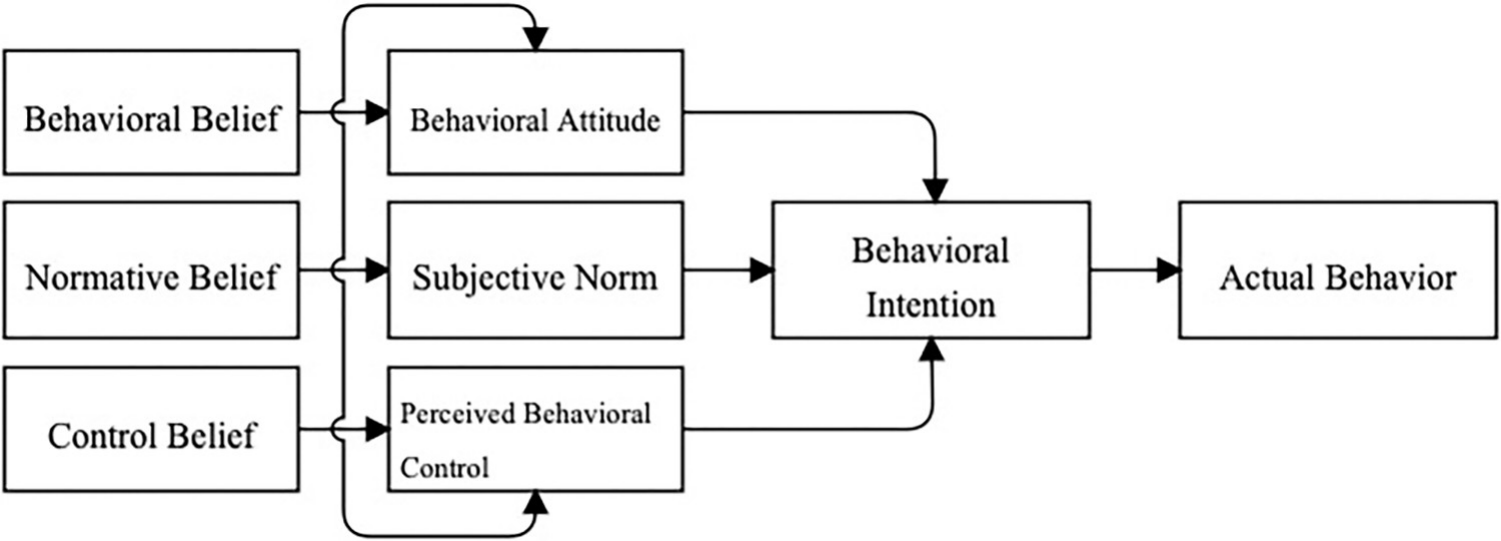
Theory of planned behavior model.

The Theory of Planned Behavior (TPB) (Fig 2), originated from Fishbein’s Theory of Reasoned Action [16], posits that behavioral intention is determined by three factors: attitude, subjective norms, and perceived behavioral control, which in turn affect behavioral attitudes. Ajzen expanded this theory by introducing perceived behavioral control, resulting in the Theory of Planned Behavior [17]. This theory comprises five key components: behavioral attitude, subjective norms, perceived behavioral control, behavioral intention, and behavior. Behavior serves as the ultimate dependent variable, while behavioral attitude, subjective norms, and perceived behavioral control generally exert effect on behavioral intention, thus affecting behavior. Accurate perceived behavioral control can sometimes directly predict the probability of behavior occurrence. Behavioral attitude refers to an individual’s subjective evaluation of their behavior and signifies their preference for specific behavioral results. Typically, a more positive attitude correlates with stronger behavioral intention and a higher probability of behavior occurrence [18]. Subjective norms represent the influence of friends, family, and societal actors when individuals respond to a particular behavior. Favorable subjective norms strengthen the intention to engage in the behavior, and conversely [19]. Perceived behavioral control denotes an individual’s perception of their level of control when engaging in a specific behavior. Homeowners with greater access to resources related to community value-added services perceive fewer obstacles and exhibit greater willingness to participate in and advocate for these services [20]. Behavioral intention reflects an individual’s inclination and motivational impetus to engage in a specific behavior, indicating the extent of their determination when intending to perform the behavior [21]. The stronger the behavioral intention, the more likely an individual is to participate.

### Research design

Upon reviewing prior research literature, it is evident that there appears to be a significant consumer concern pertaining to community medical services. Community medical services encompass comprehensive care centered on the elderly, women, children, and patients with chronic conditions within the community, combining prevention, medical care, health maintenance, rehabilitation, and health education [22]. As evidenced by He et al.’s research findings, community medical services emerge as a more cost-effective alternative to larger hospitals, effectively mitigating the financial burdens faced by patients [23]. In addition, studies by Xiao et al. highlight the simplicity of diagnostic and treatment procedures in community medical services, offering a viable solution to reduce the need for elderly patients to travel long distances for medical care [24]. Additionally, communities extend their medical capabilities beyond diagnosis and treatment, including disease prevention and rehabilitation services, with a specific focus on the elderly’s health management and the provision of personalized and comprehensive healthcare services, as indicated by Wu and Shi [25]. Wang et al. further emphasize the elderly’s desire for expanded medical services in the community, including postoperative rehabilitation, home healthcare, short-term residence, and emergency assistance [26]. These studies collectively emphasize the elderly population’s aspiration for increased community-based medical services, driven by their belief that these services enhance physical well-being and alleviate economic pressures.

Simultaneously, scholars posit that many consumers exhibit a demand for community-based lifestyle services including housekeeping services, dining, express delivery, convenience stores, and various other amenities. Community housekeeping services provide house cleaning, childcare, and care for the elderly and sick, as per the homeowner’s requests.Zhang et al. have identified homeowners’ interest in housekeeping services, noting that property management companies offering such services significantly enhance homeowners’ perception of the value-added services provided [27]. Incorporating housekeeping services in the community service framework holds the potential to boost revenue for property management companies, as indicated by Zhang [28]. Yang highlights the growing demand for community dining services due to China’s ongoing population aging, emphasizing the need to address elderly care issues [29]. In addition, Zhou introduces the concept of community group buying and express delivery operations, presenting the opportunity to reduce environmental pollution, optimize vehicle resource utilization, and minimize energy waste, thereby lowering operating costs for relevant businesses [30]. These measures also promote homeowners’ willingness towards online shopping, offering convenience in their daily lives.

With improving living standards and an increased focus on lifelong learning, community education emerges as a solution to cater to homeowners’ educational needs. Community education serves as a platform to cultivate unity, friendliness, and mutual support among individuals, concurrently enhancing their knowledge and technological capabilities to satisfy their intellectual requirements, as highlighted by Wang [31]. Robust development in community education can facilitate improved social adaptability, physical fitness, and the ability to detect fraudulent activities, thereby enhancing their sense of happiness, as proposed by Xu [32]. Correspondingly, Cheng argues that education contributes to enhancing the personal attributes of community residents by stimulating vitality, coordinating relationships, instilling values, and offering psychological counseling [33]. In an era marked by rapid technological advancements, the elderly population often experiences marginalization, leading to novel challenges and contradictions. The resultant anxiety and confusion, originating from role transitions and life changes, coupled with the social detachment experienced by the elderly, necessitate community-based elderly education initiatives, as advocated by Wang, to facilitate their transformation [34]. In summary, community education addresses residents’ spiritual needs, fosters happiness, and enables them to realize their full potential in an increasingly complex world.

With the growing demands of consumers, community customization services are poised to emerge as a significant competitive advantage. Community customized services provide tailored solutions tailored to individuals’ desires and personalized needs, aiming to boost residents’ happiness [35]. These services encompass real estate transactions, automotive services, and pet care, among others. According to the insights shared by Chen and Ye, it is advisable for communities to undertake a comprehensive analysis of individual variances including gender, age, educational attainment, economic status, living conditions, and self-care capabilities [36]. In line with each unique case, tailor-made service initiatives should be formulated to genuinely foster a sense of joy, belonging, and security among residents, thus increasing their affinity towards the community. In addition, Zhang indicates that the rise in per capita disposable income necessitates a shift towards not only universal but also personalized service provision [37]. Commencing from the personalized requisites of residents and accurately fulfilling their demands is essential for augmenting their recognition of the services offered.

**Fig 3:**
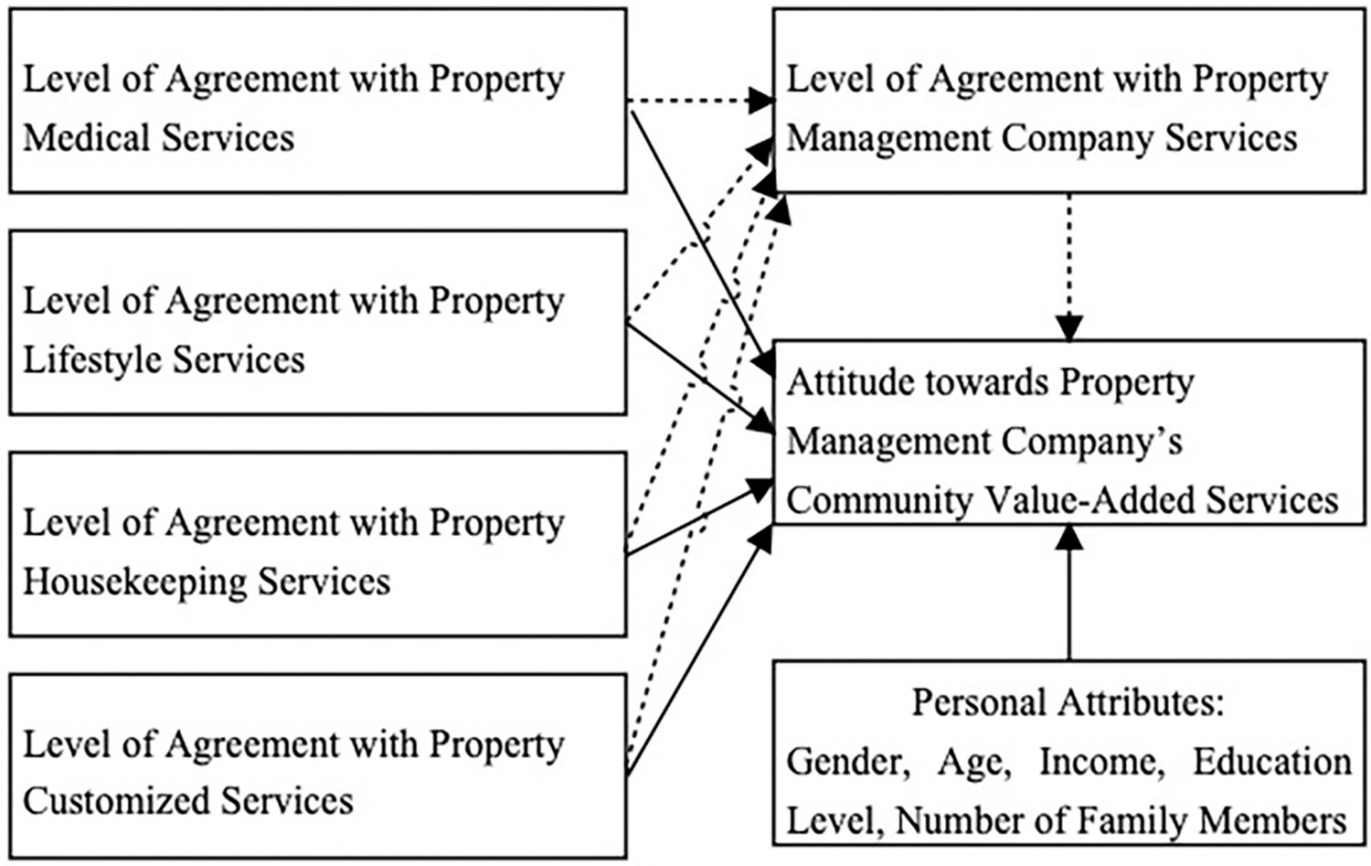
Basic framework of this study.

Based on the aforementioned literature review, a high demand among residents exists for community medical services, domestic assistance, dining facilities, educational support, spiritual solace, and personalized services. Drawing upon the perspectives of the aforementioned scholars, this study posits the following research design, grounded in the ABC theory and the theory of planned behavior. The preliminary hypothesis of this study asserts that “community medical services, lifestyle services, educational services, and customized services” affect residents’ attitudes towards property management firms delivering value-added services in the community. Moreover, individual consumer attributes may also introduce differences in the formation of consumer attitudes, thus warranting inclusion as control variables. Additionally, recognizing that the prevailing tensions between property management firms and homeowners primarily stem from homeowners’ assessment of the quality of basic services offered by property management companies, which can, in turn, affect consumer perceptions of value-added services in the community, this study incorporates homeowners’ satisfaction with the fundamental services offered by property management organizations. The objective of this study is to analyze whether homeowners’ contentment with the essential services provided by property management companies serves as a critical mediating factor in homeowners’ deliberation of embracing value-added services in the community. The preliminary design of this study is illustrated in Fig 3.

## Methods

### Data collection

Firstly, regarding the ethical review statement of this study. This research is a social survey research on consumer attitudes. The purpose of this research has been clearly stated in the questionnaire. Whether the respondents participate in the survey is voluntary, and the respondents are also anonymous to participate in the survey. The author has obtained ethical review and written approval from the Academic Committee of the School of Economics and Business Administration at Heilongjiang University.

Building upon the fundamental research framework and drawing inspiration from the questionnaire design principles advocated by relevant scholars, the questionnaire is structured around specific dimensions of value-added services. The questionnaire comprises seven sections. The first segment collects basic information from respondents, including age, gender, educational background, income, and household size. The second segment consists of a survey measuring satisfaction with services offered by the property management firm, which acts as the mediating variable in the study, and comprises five items. The third section delves into homeowners’ viewpoints concerning the property management company’s provision of value-added services, incorporating five items. The fourth section explores homeowners’ perspectives on the property management company’s delivery of medical services, including five items. The fifth segment addresses homeowners’ opinions regarding the property management company’s offering of lifestyle services, comprising five items. The sixth section tackles homeowners’ perceptions of the property management company’s provision of domestic services, comprising five items. Finally, the seventh section centers on homeowners’ views regarding the property management company’s provision of personalized and customized services, involving five items. The fourth to seventh sections of the survey primarily consider factors such as pricing, service quality, convenience, demand, and personalization concerning the envisaged services. After finalizing the questionnaire’s design, a preliminary test is administered to the primary target group to verify its rationality and ensure the questionnaire’s reliability and validity. The survey was conducted using a WeChat-based questionnaire mini program, with QR codes for accessing the survey posted in the entrances and elevators of each residential building. Additionally, a lottery mini program was incorporated at the conclusion of the questionnaire to incentivize participation. Before starting the questionnaire survey, the researcher stated the respondent’s right to decide whether to participate in the survey. Respondents can decide whether to agree to participate in the survey based on the following statement before the start of the questionnaire survey. If the respondent agrees to participate in the survey, they can start answering the questionnaire. If the respondent does not agree to participate in the survey, the survey process will be ended. Respondents decide whether to participate in the survey by checking the "Yes" or "No" dialog box in the questionnaire program. The preliminary test was conducted with 50 homeowners, and adjustments were made based on the feedback received, resulting in the final questionnaire. All questions, except those pertaining to demographics in the first section, require respondents to utilize a Likert five-point scale to express their level of agreement with different factors. Consumer attitudes were defined according to degree as “very important”, “important”, “average”, “unimportant”, and “very unimportant”. Considering the extensive representation of homeowners surveyed and the practicality of using a questionnaire, nine residential projects situated in three different provincial capital cities in China—namely Harbin, Zhengzhou, and Fuzhou—were selected as the data sources for the survey. In each region, we designated one high-end, one medium-grade, and one ordinary residential community to ensure a diverse and comprehensive sample for the survey. This study used the simple random sampling method to determine the study sample size. It was planned to survey 80 households in each community, and the overall survey sample size is 720. The formula for calculating the minimum sample size is:

$$n=\frac{P(1-P)}{\left(\frac{e^{2}}{Z^{2}}+\frac{P(1-P)}{N}\right)}$$

*P* is the standard deviation. Generally, it takes the value 0.5 when the sample variation is the largest. We hope that the survey results are within the 95% confidence interval, and the required statistic is a Z value of 1.96. We hope that the error is within 5%, and the error value represented by e is 0.05. N is the total sample size. After calculation, the minimum sample size required for this study is 251. Based on the principle of voluntary participation [38], a total of 693 questionnaires were collected in six communities, yielding 537 valid responses and resulting in a questionnaire validity rate of 77.5%. It is worth noting that the 77.5% validity rate, while slightly lower than the standard rate, was primarily due to stringent quality control measures that led to the exclusion of questionnaires with incomplete responses to specific questions. The sample characteristics are outlined in Table 1.

**Table 1:** Characteristics of the surveyed respondents.

Personal Attribute		Frequency	Percentage
**Gender**	Male	295	54.9%
	Female	242	45.1%
**Age**	Below 25 years old	24	4.4%
	25–34 years old	37	6.9%
	35–44 years old	112	20.9%
	45 years old and above	364	67.8%
**Education Level**	Secondary education	286	53.3%
	College degree	142	26.4%
	Bachelor’s degree	99	18.4%
	Graduate degree	10	1.9%
**Average Monthly Income**	Below 5000 yuan	349	65%
	5001–8000 yuan	93	17.3%
	8001–12000 yuan	59	11%
	Above 12000 yuan	36	6.7%
**Number of Household Members**	3 or fewer	346	64.4%
	3–5 people	179	33.3%
	6–8 people	8	1.5%
	8 or more people	4	0.7%

### Preliminary data analysis and hypothesis formation

Analyzing the personal attributes of the respondents reveals a significant presence of middle-aged and elderly individuals aged 45 and above, constituting a significant 67.8% of the sample. This demographic trend highlights the greater concerns regarding community services. The respondents’ educational backgrounds reflect this age-related pattern. Drawing from age and educational profiles, one can preliminarily infer that the demographic concerned with their living environment primarily comprises middle-aged and elderly individuals. Despite potential limitations in their educational attainment due to the historical era they grew up in, feedback from the questionnaire demonstrates their openness to embracing new service concepts, along with robust learning and cognitive abilities. In addition, concerning income levels, a significant 82.3% of the sampled population belongs to the category of the general population with medium to low incomes. While differences between reported and actual income may exist due to privacy concerns, the broad income intervals in the questionnaire mitigate any significant discrepancies. Nevertheless, income level emerges as a potentially influential factor shaping the demand for community value-added services, warranting further study. In the surveyed population, small families consisting of three members or individuals living independently dominate, comprising 64.4% of the total. In contrast, large families with six or more members represent a mere 2.2%. This trend indicates a prevailing preference among modern individuals for independent living, indicative of significant improvements in living conditions compared to the past. However, the prevalence of small families also signifies a robust market demand for community value-added services. Considering the typically busy schedules of family members and their limited free time, this demographic exhibits a strong demand for specific community services.

### Reliability and validity testing, factor analysis

In this research, Cronbach’s alpha coefficient served as the measure of internal consistency and reliability for the questionnaire. Typically ranging from 0 to 1, with higher values indicating greater reliability, the questionnaire demonstrated good test results, with a minimum value of 0.953. To reduce the 30 variables relevant to the research hypotheses into 6 common factors, exploratory factor analysis was conducted. SPSS 21.0 software was employed for this purpose, utilizing principal component analysis with eigenvalues exceeding 1 and varimax rotation. Before conducting factor analysis, the Keiser-Meyer-Olkin (KMO) test and Bartlett’s sphericity test were executed to measure the suitability of the questionnaire data for factor analysis. Factor analysis was executed independently for six discrete sections, representing the independent variables (Sections 4, 5, 6, and 7 of the questionnaire), mediating variables (Section 2 of the questionnaire), and dependent variables (Section 3 of the questionnaire). The results of the factor analysis are presented in Table 2.

**Table 2:** Exploratory factor analysis of the questionnaire.

Factor Group	Question	Cronbach’s α	KMO Test	Bartlett’s Test	
				X^2^	Sig.
Identification of Property Company Services	6,7,8,9,10	.954	.886	2998.834	.000
Attitude towards Property Company’s Community Value-Added Services (dependent variable)	11,12,13,14,15	.953	.886	2884.640	.000
Identification of Property Medical Services	16,17,18,19,20	.973	.923	3940.688	.000
Identification of Property Lifestyle Services	21,22,23,24,25	.966	.908	3525.227	.000
Identification of Property Educational Services	26,27,28,29,30	.979	.919	4424.207	.000
Identification of Property Customized Services	31,32,33,34,35	.977	.917	4152.536	.000

#### Hypotheses proposal

*Community medical services*. After three years of the pandemic, the explicit needs of individuals have presently shifted towards medical care and emotional support, while implicit needs consist of home care and rehabilitation nursing [39]. And China is facing a serious aging population, with an increasing number of elderly people and large hospitals in short supply. Therefore, With the gradual implementation of the “major illnesses to hospitals, minor illnesses to the community” two-way referral system, there has been an increasing influx of patients at community health service centers [40]. In addition, community medical services is an important guarantee for improving the health level of residents, which is convenient for medical treatment, reduces the cost and time of diagnosis and treatment of patients, and can meet the growing demand for community medical services of residents [41].To better align with residents’ demands and augment revenue, it has become an inevitable trend for property management companies to offer community medical services, integrate community medical resources, and elevate the caliber of community healthcare. In light of this, the subsequent hypothesis is posited:

H1: The residents’ recognition of medical services has a positive and significant impact on the property management company’s provision of community value-added services.

*Community lifestyle services*. Amid the rise in people’s material affluence and increasingly hectic work schedules, a majority tend to economize time by procuring groceries and daily essentials locally and engaging housekeep help for household chores [12]. Nevertheless, sourcing products externally and hiring external personnel often involves variable quality and higher costs. Property management services are proactively diversifying into community lifestyle amenities to address societal requisites and fulfill residents’ needs [42]. Delivering consistent quality, cost-effectiveness, and time efficiency in services can augment residents’ appreciation of community value-added amenities. Building on the preceding analysis, the subsequent hypothesis is formulated:

H2: The residents’ recognition of lifestyle services has a positive and significant impact on the property management company’s provision of community value-added services.

*Community education services*. The concept of lifelong learning is steadily gaining traction, with residents actively seeking educational opportunities that align with their needs and address their questions and concerns. Owing to demanding work commitments, time constraints, safety considerations, and health constraints, residents favor educational institutions in proximity to their residences that offer high-quality education. Community education aspires to positively affect the physical and mental well-being of community dwellers, enhance their personal attributes and living standards, promote lifelong advancement, and contribute to the sustainable development of the community [43, 44]. In the future progression of property community services, offering community education services to cater to residents’ requisites can bolster their appreciation of community value-added amenities. Based on the above analysis, the following hypothesis is proposed:

H3: The residents’ recognition of community education services has a positive and significant impact on the property management company’s provision of community value-added services.

*Community customized services*. As society evolves and individuals’ purchasing power rises, there is an observable trend towards diversification and personalization in the demand for service attributes. Numerous businesses seek to bolster their competitive standing in the market by offering distinctively personalized services [45, 46]. To improve residents’ satisfaction with property services, property management needs to consider the heterogeneity of different residents’ preferences and personalized needs. Personalized needs are the self-awareness that everyone has, the desire to pursue uniqueness. When individuals feel their uniqueness is threatened, they may restore self-esteem and reduce negative impacts by pursuing differentiation [35]. Specifically, individuals may enhance their social image by acquiring and using specific items to differentiate themselves from others [47]. Therefore, community customized services should be based on each person’s innate self-awareness and desire to pursue uniqueness [35], providing personalized customized services for residents and thereby improving the satisfaction of property owners with property services. It can be seen that providing customized services in the community will bring significant advantages. Through the provision of bespoke services, property management organizations have the capacity to address the unique needs of individual residents, thereby augmenting their overall satisfaction with property-related services. Thus, the following hypothesis is proposed:

H4: The residents’ recognition of community customized services has a positive and significant impact on the property management company’s provision of community value-added services.

*Mediating effect of basic service satisfaction*. In reality, the service quality of property management companies needs to be improved. The conflict between property owners and property management companies in basic services such as parking, cleaning, and security is becoming increasingly severe, which not only seriously affects the lives and emotions of property owners, but also leads to the deterioration of the operating conditions of property management companies [48]. Therefore, owner satisfaction is becoming increasingly important. Property management companies can only gain the support of property owners by providing property services based on their satisfaction. This is the only way to ensure the continuous operation of the company [3, 49]. Just as consumers’ identification with manufacturers greatly affects their attitude and willingness to purchase goods during the process of selecting them. The satisfaction of property owners with the basic services provided by the property management company will also have an impact on whether they agree with the company’s community value-added services. Therefore, the following hypothesis is advanced:

H5: Satisfaction with property management services plays a mediating role in the homeowners’ recognition of the added value services provided by property management companies.

## Results

### The impact of personal attributes on consumer cognition

To clarify the differences in residents’ perspectives regarding the delivery of community value-added services by property management companies, this study employed independent sample t-tests and one-way ANOVA to appraise the effect of different individual attributes on residents’ attitudes. The findings are detailed in Table 3.

**Table 3:** Influence of individual attributes on residents’ attitudes towards property management companies’ provision of community value-added services.

Dependent Variable	N	Mean	Standard Deviation	Attribute Variable	F	Sig.
Attitude towards Property Company’s Community Value-Added Services	537	4.34	.831	Gender	.150	.699
				Age	4.68	**.003**
				Education Level	1.578	.194
				Monthly Income	2.684	**.046**
				Cohabitants	1.639	.179

From the test results, it can be observed that the various characteristics of the residents involved exert different effects on their perspectives regarding the provision of community value-added services by property management companies. Levene’s test results for gender reveal no significant divergence in attitudes towards property management companies’ provision of community value-added services based on gender. In addition, the one-way ANOVA results for education level and the number of cohabitants in the survey yield p-values of 0.194 and 0.179, respectively, which do not reach significance at the 95% confidence level. This signifies that neither education level nor the number of cohabitants significantly affects residents’ attitudes towards property management companies’ provision of community value-added services. However, the one-way ANOVA findings for age and monthly income of the surveyed residents attain significance at the 95% confidence level, suggesting significant differences in attitudes towards property management companies’ provision of community value-added services among residents of varying income levels and age groups. These findings emphasize the growing awareness among people concerning their demand for service quality, irrespective of their educational background. Additionally, regardless of household size, people generally prioritize the comfort of their living environment. These two points emphasize the increasing importance individuals attribute to quality-of-life services today, highlighting the vast potential market for community value-added services.

However, the results also reveal certain distinctions in the surveyed residents’ attitudes towards property management companies’ provision of community value-added services due to differences in age and monthly income level. In summary, this finally arises from the effect of the pricing nature of most value-added services on different income brackets. For low-income households, while they possess a psychological desire for property value-added services and practical needs for these services, income indeed plays a crucial role in determining whether this group can finally afford value-added services. Hence, property management companies should thoughtfully contemplate pricing standards for value-added services prior to entering the market and formulate a rational pricing system for value-added services, considering regional income conditions. Additionally, the age-related differences are related to the consumption behavior of various age groups. With age, social experience grows, and the perception of novelty becomes more rational. Middle-aged and elderly individuals typically embrace a thrifty and economical attitude towards life, which aligns with the evolving societal dynamics in China. When it comes to non-essential expenses, middle-aged and elderly individuals typically prefer self-sufficiency. Particularly in the context of community value-added services, which are not essential for basic living but enhance the quality of life, they often prioritize thriftiness over consumer awareness, which may affect their decision to engage with value-added services. In reality, property management companies can contemplate incorporating some low-cost and widely accessible complimentary value-added services when introducing community value-added service systems. This enables the middle-aged and elderly population to experience the convenience and enhancement in quality of life provided by community value-added services, gradually reshaping their traditional consumption patterns and gradually embracing this novel approach to improving their quality of life.

Based on the aforementioned research findings, future analyses will consider age and monthly income as control variables when assessing their effect on residents’ attitudes towards property management companies’ provision of community value-added services, while also considering other independent variables.

### Results of hypotheses test

Based on the research hypothesis, following an analysis of residents’ attitudes toward property management companies’ provision of community value-added services based on their personal characteristics, a hypothesis test was conducted to assess the impact of residents’ recognition of community medical, daily life, education, and customized services on their attitudes toward property management companies’ provision of such services. Control variables, namely residents’ age and average monthly income, were incorporated into the test equation. Before conducting the regression analysis, both control variables, age and average monthly income, were converted into dummy variables. The results of the regression analysis are presented in Table 4.

**Table 4:** Regression analysis of the impact of residents’ attitudes towards property management companies’ provision of community value-added services.

Hypothesis	VIF	*β*	Ad *R*^2^	Standard Error	P	Confidence Interval	Results
H1	7.134	.666***	.747	.028	0.000	[4.07869,4.25129]	Supported
H2	8.772	.680***	.772	.032	0.000	[4.16269,4.31702]	Supported
H3	9.777	.653***	.777	.031	0.000	[4.16678,4.32707]	Supported
H4	7.136	.474***	.658	.028	0.000	[4.04891,4.23266]	Supported

^a^Dependent variable: Attitude towards Property Company’s Community Value-Added Services; ^b^***. Significantly correlated at the 0.01 level. ^c^N = 537

The regression results indicate a significant association between residents’ attitudes toward medical, daily life, education, and customized services and their perception of property management companies’ provision of community value-added services. The regression model demonstrates a strong fit and effectively explains the effect of various services on residents’ attitudes toward property management companies’ enhancement of community value-added services. The significance level of residents’ recognition of these four services, in enhancing their acceptance of property management companies’ community value-added services, is 0.000, signifying that these services substantially boost residents’ acceptance of such services.

### Mediation effects of property service satisfaction

In practice, numerous property management companies grapple with significant conflicts with residents, largely attributed to residents’ dissatisfaction and lack of acknowledgment of the fundamental services rendered by these companies, leading to decreased resident satisfaction levels. Resident satisfaction is a variable with far-reaching impact. Low satisfaction not only results in unstable income for property management companies but, more crucially, erodes residents’ trust in these companies. Without trust, residents are unlikely to recognize other initiatives undertaken by property management companies. This survey includes a specific dimension of "property service satisfaction" to explore its mediating effect on residents’ attitudes toward property management companies’ provision of community value-added services.

Employing the Bootstrap sampling method [50], the empirical findings regarding the mediation effect of "property service satisfaction" are presented in Table 5.

**Table 5:** Mediation effect of “Property service satisfaction”.

Dimension Examined	Effect Type	Effect	SE	p	Boot LLCI	Boot ULCI	Proportion
Attitude towards Medical Services	Total Effect	.708	.018	.000	.672	.744	-
	Direct Effect	.579	.023	.000	.534	.623	81.78%
	Indirect Effect of Property Service Satisfaction	.129	.030		.073	.190	18.22%
Attitude towards Daily Life Services	Total Effect	.805	.019	.000	.767	.843	-
	Direct Effect	.674	.025	.000	.626	.722	83.73%
	Indirect Effect of Property Service Satisfaction	.131	.041		.057	.219	16.27%
Attitude towards Community Education Services	Total Effect	.776	.018	.000	.739	.812	-
	Direct Effect	.642	.022	.000	.598	.685	82.73%
	Indirect Effect of Property Service Satisfaction	.134	.035		.071	.209	17.27%
Attitude towards Customized Services	Total Effect	.619	.020	.000	.580	.658	-
	Direct Effect	.463	.022	.000	.419	.507	74.8%
	Indirect Effect of Property Service Satisfaction	.156	.032		.097	.222	25.2%

According to the test results, Within the 95% confidence interval for estimating the mediation effect, interval estimates do not include 0. This suggests that property service satisfaction serves as a mediator in residents’ attitudes toward property management companies’ provision of community value-added services. In essence, residents assess the quality of property management companies’ basic services while considering the acceptance of these services.

## Discussion

In the post-pandemic era, there has been a significant increase in consumer demand for medical services. However, due to policy influences, time constraints, complex hospital procedures, transportation difficulties to hospitals, and high medical expenses, many residents hesitate to seek medical treatment in hospitals [51]. Therefore, offering medical services in property communities can significantly alleviate these issues and effectively cater to residents’ needs. Hence, property management companies’ provision of medical services has a positive and significant effect on consumers’ recognition of community value-added services.

With mounting work-related pressures and an increasing focus on quality of life, there is a growing desire among individuals for convenient access to various services, such as housekeeping, dining, and convenience stores. However, when consumers attempt to obtain these services externally, they often encounter challenges such as inconsistent quality, inadequate security assurances, and significant time investments. Therefore, the availability of life services in communities proves beneficial in enhancing residents’ quality of life and fostering community cohesion. Therefore, this significantly enhances users’ perception of community value-added services.

As the idea of lifelong learning gains increasing popularity, the government places significant emphasis on the enhancement of community education services. Several policy documents have been introduced to promote the establishment of these services. Moreover, there is a growing demand among community residents for more diverse and personalized learning experiences. Homeowners aspire to have convenient access to comprehensive educational services in close proximity. Therefore, community education services offered by property management companies are positioned to cater to consumer needs. Hence, the provision of such community education services yields a positive and significant effect on consumers’ perception of the value-added services in their community.

In light of societal advancements and the diversification of consumer preferences, the necessity for community services has become increasingly personalized. Traditional standardized services are no longer sufficient to meet the requirements of all residents. Additionally, advancements in technologies such as the Internet and big data have made it possible to offer customized services in communities. Through these technological means, communities can gain a more accurate understanding of residents’ needs and deliver personalized services. Satisfaction and contentment can only be enhanced by deeply understanding and accurately addressing consumer demands, thereby strengthening residents’ sense of community identity and belonging. Therefore, the provision of customized community services exerts a positive and significant effect on residents’ perception of community value-added services.

When a property management company excels in its core services, homeowners will correspondingly exhibit high levels of satisfaction and trust in the company. In such cases, homeowners will also exhibit a positive attitude towards the community’s value-added services. Conversely, inadequate performance in core property services by the property management company results in low homeowner satisfaction and a lack of trust and recognition toward the company. In such instances, the provision of value-added community services will further exacerbate homeowners’ distrust towards the property management company. This highlights the importance of property management companies enhancing their basic property services during the early stages to boost homeowner satisfaction. Building a strong mutual trust with homeowners through the delivery of core services is crucial for fostering the development of value-added services.

## Conclusion

The attitude of homeowners towards property management companies offering community value-added services vary based on factors such as age and household income. Therefore, in the early stages of a project, companies should conduct in-depth market research and design a comprehensive business plan that fully takes into account the effect of individual attributes. Targeted strategies should be proposed to strengthen homeowners’ understanding and appreciation of community value-added services. For instance, during the initial promotional phase, emphasis can be placed on the convenience and safety of services, as well as the provision of differentiated services catering to the diverse needs of various age groups and income levels. In addition, establishing consumer perceptions is critical, as promotion alone may not be as effective as firsthand experiences. Thus, investment in enhancing homeowners’ experiences should be considered.

Empirical research has been carried out to assess consumer attitudes towards community value-added services, and the findings indicate that all five principal factors exert a significant positive effect on consumers’ willingness to engage in such services in the community. Community medical services, community life services, community education services, and community customized services are all marketable and enjoy widespread recognition among homeowners, thereby fostering their acceptance of property management companies’ systematic community value-added services. Should homeowner satisfaction be further enhanced through business operations, it will play a more constructive role in advancing the development of additional community value-added services. This necessitates property management companies to assess their own strengths and available resources when contemplating pilot projects. They should select specific or partial services based on targeted market research, accumulate experience in the practical process, emphasize service quality, progressively enhance homeowner satisfaction, and secure recognition for the community value-added services provided by property management companies. In conclusion, property management firms are strategizing to leverage the potential of community value-added services, innovate their service models and products, extend the property service continuum, and offer homeowners with a comfortable and convenient lifestyle, thereby expanding the profit potential of the company and introducing fresh opportunities for development. Nonetheless, property management companies must carefully weigh their choices in the development of community value-added services, taking into account their specific circumstances and available resources, while avoiding depletion of manpower and resources.

As homeowner satisfaction with property services exerts a mediating effect on homeowners’ perceptions of property management companies’ delivery of community value-added services, property management organizations must remain mindful of their core mission, diligently execute their foundational property service duties, and strive for continuous improvement in homeowner satisfaction. Only through these means can mutual trust be cultivated between the company and homeowners, fostering homeowners’ acknowledgment and backing for the company’s provision of community value-added services.

However, there remain several limitations and inadequacies in this study. Firstly, the research scope is confined. The survey respondents in this study were subject to certain restrictions, and the questionnaire may yield different results in cities characterized by slower economic development and lower educational levels. Secondly, the criteria for service quality remain somewhat unclear. Different consumers, identified by their varying income levels and educational backgrounds, hold different criteria for quality. However, this aspect was not factored into the survey, and the quality standards in the questionnaire were contingent on consumer perceptions. In future efforts toward establishment and implementation, more exhaustive research and analysis should be conducted.

This study offers two primary contributions. Firstly, it caters to consumer demands. By surveying homeowners, property management firms can identify their requirements and enhance the supply process to better align with consumer needs. Secondly, it aligns with societal progress. In the contemporary context of high work pressure, extended work hours, and deeper educational attainment, traditional property services no longer suffice to meet consumer demands. Firms must adapt to societal evolution and vigorously cultivate value-added services to sustain profitability.

## Supporting information

S1 File Data. (XLSX)
